# Evaluating Multi-Benefit Cover Crop Management Models for Citrus Sustainable Management: A Field Study from Central China

**DOI:** 10.3390/plants15101479

**Published:** 2026-05-12

**Authors:** Rong-Bin Tang, Li-Juan Li, Yin-Hua Guo, Rui Yuan, Yu-Tong Feng, Jun-Chen Wang, Yun-Chao Yu, Hao-Yong Song, Jun Zhang, Di Wu, Gan-Ju Xiang

**Affiliations:** 1 Hubei Key Laboratory of Rare Resource Plants in Three Gorges Reservoir Area, China Three Gorges Corporation, Yichang 443100, China; tangrongbin126@126.com (R.-B.T.); lilijuan18@mails.ucas.ac.cn (L.-J.L.); guo_yinhua@126.com (Y.-H.G.); yuan_rui3@ctg.com.cn (R.Y.); 17771712468@163.com (Y.-T.F.); wang_junchen@ctg.com.cn (J.-C.W.); yu_yunchao@ctg.com.cn (Y.-C.Y.); song_haoyong@ctg.com.cn (H.-Y.S.); zhang_jun15@ctg.com.cn (J.Z.); wu_di3@ctg.com.cn (D.W.); 2 National Engineering Research Center of Eco-Environment in the Yangtze River Economic Belt, China Three Gorges Corporation, Beijing 100083, China

**Keywords:** citrus, cover crops, CO_2_ flux, leaf physiological traits, soil properties, sustainable management

## Abstract

Cover crop in orchards is recognized as a sustainable practice that enhances multiple ecosystem services, yet systematic evaluations of different cover crop management models in citrus orchards remain limited. This study investigated the effects of cover crop management models (natural cover crop: T1, *Lolium perenne* L.: T2, *Trifolium repens* L.: T3, *Vicia villosa* Roth: T4, and mixed cover crops: T5) on soil properties, soil CO_2_ flux, leaf physiological traits, fruit quality, and yield in a citrus orchard, using clean tillage as a control. Results showed that cover crop management models significantly influenced soil water content, available nitrogen (AN), available phosphorus (AP), and available potassium (AK). The *V. villosa* model (T4) reduced AN and AP but enhanced leaf chlorophyll (Cl) and nitrogen (N) content. Soil CO_2_ flux was significantly higher under T4, and it showed the lowest soil moisture. The results of mantel tests revealed that AP and soil moisture were key drivers of leaf traits, though no significant treatment effects on fruit quality or yield were detected within the two-year experimental period. These findings indicate that cover crop management models rapidly alter soil properties and CO_2_ emissions, but longer-term observations are needed to evaluate cascading effects on fruit. This study offers evidence-based soil management solutions and a framework for enhancing multiple ecosystem services in orchards worldwide.

## 1. Introduction

The global temperate and subtropical management approaches of orchard ecosystems are experiencing a significant transformation [1,2]. The conventional intensive clean-tillage system is focused on enhancing short-term operational efficiency [3], but it has progressively led to significant ecological consequences, including accelerated mineralization of soil organic matter, reduced microbial community complexity, impaired nutrient cycling, and increased vulnerability to soil erosion [3,4,5]. These adverse effects not only compromise the long-term productivity of orchard systems but also weaken the resilience of agricultural ecosystems under external environmental pressures [6,7]. At the same time, nature-based solutions have gathered growing recognition [8,9]. Among these, cover crops in orchards have emerged as a sustainable practice that concurrently enhances agricultural output and ecological functionality and is increasingly implemented worldwide [10,11]. Moreover, it aligns with the United Nations’ Sustainable Development Goals related to soil health and terrestrial ecosystem conservation [12] and further supports global efforts toward carbon neutrality through enhanced agricultural carbon sequestration [13].

Cover crop management models, as a living ground cover, have been proven to have multiple ecological benefits through the establishment of specific plant communities among tree rows in orchards [7,14,15]. A well-configured grassing system can significantly enhance the organic carbon content of surface soil through litter return, root turnover, and microbially driven transformation processes, thereby improving the structure and water stability of soil aggregates [16,17,18]. In terms of nutrient management, the inclusion of Fabaceae species can reduce dependence on chemical nitrogen fertilizers through biological nitrogen fixation and facilitate the mobilization of insoluble phosphorus and potassium in the soil, thereby optimizing the spatial–temporal alignment between nutrient supply and fruit tree demand [11,19,20,21,22]. Furthermore, the cover crop layer can achieve ecological regulation of the orchard weed community by modulating the near-surface microclimate, competing for ecological niches, releasing allelochemicals, and suppressing weeds, thereby reducing reliance on herbicides [23,24,25,26]. From the perspective of ecosystem service functions, a diverse cover crop can provide habitats for natural enemy insects and soil fauna, enhance biological control capacity [27,28], and reduce the risk of nitrogen leaching and runoff pollution associated with exposed soil [14,29,30,31].

In the context of global climate change, soil surface cover types are critical regulators of soil CO_2_ flux (soil respiration), which directly governs ecosystem carbon cycling and greenhouse gas emissions. Studies have confirmed that different ground cover regimes (e.g., clean tillage, cover crop, straw) create distinct soil hydrothermal conditions and microbial habitats, leading to significant variations in CO_2_ emission rates and annual fluxes [32]. Compared with bare soil or clean tillage, perennial cover crop generally elevates soil CO_2_ flux by enhancing root exudation, stimulating microbial decomposition, and improving soil porosity and moisture retention; however, certain cover types can suppress CO_2_ emissions by buffering soil temperature fluctuations and reducing organic matter mineralization in hot seasons [33,34]. These divergent effects highlight the necessity of optimizing cover composition to balance carbon sequestration and greenhouse gas mitigation in orchards.

Citrus is the most widely produced fruit crop worldwide [35]. According to China Agricultural Statistical Data, China has ranked first globally in both citrus cultivation area and output since 2007, surpassing Brazil [36]. Currently, growers primarily select cover crop species based on empirical knowledge or single functional traits, such as soil and water conservation, leading to the lack of a systematic, multidimensional evaluation that integrates ecological benefits (e.g., soil health and biodiversity), economic returns (e.g., reduced management costs and increased yields), and environmental services (e.g., carbon sequestration and erosion control). Consequently, the demand among citrus growers for low-competitiveness, high-benefit cover crop species remains largely unmet [37].

Some researchers have intensified efforts to breed low-competitive cover crop species and optimize their configuration models [31,38]. For instance, several institutions have initiated systematic screening programs and field-based adaptability assessments for cover crop species adapted to citrus orchard environments. Evidence from preliminary studies demonstrates that *Trifolium repens* L., *Vicia villosa* Roth, *Lolium perenne* L., and a *T. repens*–perennial cover crop mixture all demonstrated high weed suppression coverage; *L. perenne* significantly improved soil porosity, while Fabaceae covers distinctly enhanced soil organic matter content [21]. *L. perenne* and *V. villosa* significantly enhanced the vegetative growth of citrus trees and effectively reduced surface runoff as well as nitrogen and phosphorus losses [39]. Full-cover crops performed better than strip-cover crops in both *Paspalum notatum* Flugge and *Cnodon dactylon* (L.) Persoon [40]. Compared with natural pasture, pasture Fabaceae alone, Poaceae alone, and a mixture of Fabaceae and Poaceae demonstrated greater efficacy in mitigating water and soil losses as well as associated nitrogen and phosphorus losses [40]. The combined use of Fabaceae and non-Fabaceae cover crops can effectively increase the nitrogen transformation rate in orchard surface soils and reduce greenhouse gas emissions by promoting the abundance of nitrous oxide-reducing bacteria, thereby enhancing soil microbial functions. Nevertheless, while Poaceae coverage, (e.g., *P. notatum*), Fabaceae (e.g., *V. villosa*, *T. repens*), and creeping species (e.g., *Dichondra repens* Forst.) hold promise for enhancing ecosystem multifunctionality, robust empirical data are still lacking on which specific combinations can simultaneously optimize ecological integrity, economic efficiency, and environmental sustainability in citrus orchard systems. To our knowledge, large-scale and systematic screening studies on cover crop management in citrus orchards remain scarce, particularly those involving comprehensive comparative assessments of soil quality, fruit quality, and carbon sequestration potential.

In this study, we conducted one field experiment to systematically compare diverse cover crop species and their configuration patterns in Xingshan County, Yi Chang City, Hubei Province. This region is recognized as a globally significant citrus-producing region due to its typical subtropical monsoon climate and widespread issues of red and yellow soil acidification and compaction [41,42,43]. Orchards in this region are undergoing a critical transition from high-yield-oriented practices to high-quality, environmentally sustainable production systems, creating an urgent practical need to develop optimized, site-adapted cover crop management models [39,44]. Specifically, we addressed three research questions: (i) the distinct improvement pathways of key soil properties under different cover crop management models; (ii) the causal relationships between improved soil conditions and fruit quality (including soluble solids, total acidity, vitamin C, and flavonoid content) as well as commercial appearance; and (iii) soil respiration rates and net carbon balance across contrasting cover crop systems. This study will help identify low-competitiveness, multi-benefit cover crop species and optimal configuration strategies for citrus orchards. By linking soil improvement to fruit quality and carbon dynamics, the findings provide evidence-based solutions for sustainable soil management and a framework for enhancing multiple ecosystem services in orchard systems, with implications for analogous agroecological regions worldwide.

## 2. Materials and Methods

### 2.1. Study Site and Environmental Conditions

**Figure 1:**
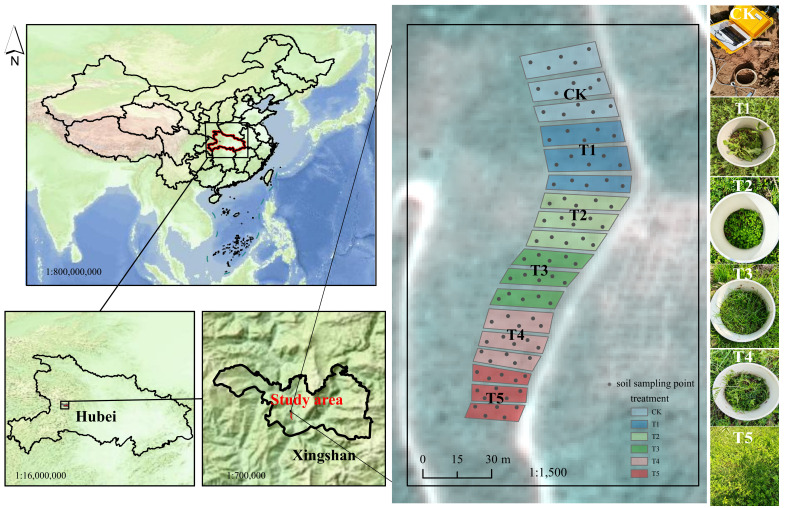
The regional location and the treatment in this study. CK, clean tillage; T1, natural cover crop; T2, Poaceae cover crop (*Lolium perenne* L.); T3, Fabaceae cover crop (*Trifolium repens* L.); T4, Fabaceae cover crop (*Vicia villosa* Roth); T5, mixed cover crop combination (*Astragalus sinicus* L., *L. perenne*, *T. repens*, *V. villosa* and *Cynodon dactylon* (L.) Persoon.).

Study plots were established within a hilly citrus orchard in southwestern Hubei Province, central China (110.7801° E, 31.1326° N; Figure 1). This region has a subtropical monsoon climate [45], with a mean annual temperature of 22.6 °C and an average annual precipitation of 1574 mm. The climate is characterized by concurrent rainfall and heat, with a frost-free period exceeding 350 days. According to international classification systems [46], the soil type is classified as red soil. The results of the soil baseline samples collected before the start of the cover crops experiment in 2023 are as follows: soil moisture content 0.83%, pH 5.12, organic matter content 21.52 g/kg, total nitrogen content 1.45 g/kg, total phosphorus content 0.75 g/kg, total potassium content 7.56 g/kg, available phosphorus content 89.38 mg/kg, and available potassium content 239 mg/kg. According to local meteorological station records, the annual average temperature during the experimental period ranged from 16.2 °C to 17.5 °C Monthly mean temperatures ranged from 4.8 °C (January) to 28.6 °C (July–August). Annual cumulative precipitation ranged from 980 mm to 1250 mm, with over 75% concentrated in the growing season (April to October). These conditions are consistent with the local long-term climate and provided stable environmental conditions for the field trial.

### 2.2. Experimental Design and Treatment

Five- to eight-year-old Lane Late navel orange (*Citrus sinensis* (L.) Osbeck cv. Lunwan) trees were planted at a spacing of 4 m × 2 m, with an average canopy height of 2 m and vigorous growth. The cover crop seeds used in the trial were commercially obtained, with a purity greater than 99% and a germination rate exceeding 95%.

The experiment included six treatments, each with three biological replicates. The treatments were as follows: clean tillage (CK), natural cover crop (T1), Poaceae cover crop with *Lolium perenne* L. (T2), Fabaceae cover crop with *Trifolium repens* L. (T3), Fabaceae cover crop with *Vicia villosa* Roth (T4), and a mixed cover crop combination (T5) including common cover crop species such as *Astragalus sinicus, L. perenne*, *T. repens*, *V. villosa* and *Cynodon dactylon*. The range of species and functional groups reflects those that are known to be successful in restoration schemes and occur across a wide range of mesotrophic grassland types in this zone. To control the risk of cross-dispersal and varietal contamination among different cover crop species, a treatment strip design was adopted. Six treatments were established, each with three replicate plots. All 18 plots were spatially isolated from one another to prevent edge effects and species mixing. The three replicates of each treatment were arranged continuously along the contour lines to form independent treatment strips, with a blank area placed between adjacent strips. To minimize the influence of soil heterogeneity across the slope, each treatment strip was extended along the slope direction, covering an area of approximately 90–100 m^2^. All strips were located within an area characterized by consistent topography, slope gradient, and initial soil conditions. The three replicates of all treatments were concentrated within the area shown in Figure 1, and the complete experimental layout, including spatial separation and replicate distribution, is illustrated in Figure 1. The citrus orchard was managed under standard commercial practices. All treatments received the same irrigation regime (frequency, duration, and volume), ensuring that irrigation did not confound the evaluation of cover crop effects on soil properties. Cover crop management was maintained over a two-year period.

Prior to the experiment, the orchard was cleared of stones, unwanted trees, and noxious weeds, and the soil was plowed to a depth of 10–15 cm. Soil management across the entire orchard was uniformly maintained under clean tillage and manual weeding before treatment initiation. In the CK treatment, all weeds within rows were removed manually on a regular basis, with weeding frequency adjusted according to field conditions. In T1, no weeding was conducted, allowing naturally occurring herbaceous plants to grow freely throughout the plot. For treatments T2, T3, T4, and T5, seeds were broadcast in the inter-row zones at a distance of 50 cm from citrus tree trunks. Seeding rates were approximately 1.0–1.5 kg/ha for Trifolium repens and *V. villosa* and 2.5–3.0 kg/ha for *L. perenne* and *A. sinicus*. After sowing, the soil surface was lightly raked to ensure adequate seed coverage. It is particularly noteworthy that T5 was established by sowing various cover crop species in equal proportions. Based on field observations, all species grew uniformly, and no single species dominated the mixture.

### 2.3. Data Collection and Processing

The experiment ran from September 2023 to October 2025. In September 2023, the field was tilled and plots were demarcated, followed by cover crop sowing in October 2023. In April 2025 (peak growing season), soil carbon flux monitoring, leaf sampling, and citrus yield recording were conducted. In September 2025, after cover crop decomposition, soil samples were collected for chemical analysis. No additional tillage or reseeding was performed, so cover crop management continued uninterrupted over the two-year period.

#### 2.3.1. Soil Assessments

Soil sampling was performed in September 2025 after cover crops’ decomposition. Within each of the three replicate plots per treatment, five representative points were selected following an S-shaped pattern, and surface soil samples (0–20 cm) were collected and thoroughly homogenized to form 1 composite sample per replicate plot (meaning 3 composite samples per treatment and 18 in total). The spatial distribution of sampling points within each plot is illustrated in Figure 1.

For each composite sample, one subsample was sealed in an aluminum container for laboratory determination of moisture content via the oven-drying method; another subsample was manually cleared of impurities (e.g., stones and root fragments), air-dried under ambient conditions, and sieved through a 2 mm mesh for analysis of physicochemical properties. Selected chemical properties of soil were analyzed according to these methods as follows.

In September 2025, mixed soil samples (0–20 cm) from five spots in each plot were obtained using an auger after the removal of impurities on the surface. The collected soil samples were air-dried, ground, and then partly passed through 2.0 mm sieve and partly through a 0.25 mm sieve. The sieved and dried soil samples were analyzed for rate of water content (RWC, %), total nitrogen (TN, g/kg), total phosphorus (TP, g/kg), total potassium (TK, g/kg), organic matter (OM, g/kg), available nitrogen (AN, mg/kg), available phosphorus (AP, mg/kg), available potassium (AK, mg/kg), and pH value. All soil chemical properties were analyzed following the procedures described in [47,48,49,50]. Specifically, soil pH was determined using a suspension of the soil sample in water at a ratio of 1:2.5 (*w*/*v*) with a Mettler Toledo 320 pH meter; available phosphorus was extracted by the Olsen method; and available potassium was extracted with ammonium acetate and determined by atomic absorption spectrophotometry. Available nitrogen was determined by the alkaline hydrolysis diffusion method, as detailed in the aforementioned reference.

In situ measurements of soil CO_2_ flux were taken using a portable soil carbon flux system (PS9000; Lijia United, Beijing, China). According to the manufacturer, the instrument provides a reading accuracy of approximately ±1.5%, making it appropriate for ecosystem-level CO_2_ flux monitoring [51]. Three permanent PVC collars (20 cm inner diameter, 15 cm height) were placed in each plot to serve as measurement bases. Each collar was inserted 12 cm into the soil, leaving 3 cm above the ground to reduce lateral gas escape and ensure a stable seal between the chamber and collar. This insertion depth is particularly critical in high-altitude ecosystems, where fine roots are mainly located in the top 0–10 cm soil layer; thus, the insertion effectively severs most living roots inside the collar, thereby reducing the contribution of autotrophic (live root) respiration to the measured CO_2_ flux. The collars were installed on April 18, 2025, and the first CO_2_ flux measurements were taken on April 30, 2025, allowing sufficient time for installation disturbances to subside. Throughout the monitoring period, fieldwork followed a consistent schedule and standardized protocol. For each measurement, the automated chamber was sealed onto the collar, and the increase in CO_2_ concentration was recorded over a brief closure period (typically 5–10 min). During measurement session, the device logged CO_2_ concentrations within the chamber, chamber temperature (CT, ℃), atmospheric pressure (Apa, kPa), soil temperature (ST, ℃), soil moisture (SM, %), and average H_2_O content (ppm) in real time. Using the system’s software, the initial stabilization period after chamber closure and any apparent outliers in the CO_2_ time series were excluded [52]. CO_2_ flux was then calculated based on the linear rate of CO_2_ concentration change inside the chamber, the chamber volume, and the soil surface area enclosed by the collar, with results expressed in umol/(m^2^*s).

#### 2.3.2. Fruit Quality Assessment and Yield Statistics

Leaf chlorophyll content (as an indicator of nitrogen status) was rapidly assessed during the vigorous growth period using a chlorophyll meter (model IN-YL03; Shandong Laiyin Optoelectronic Technology Co., Ltd., Tsingtao, China). Chlorophyll meter readings (SPAD values) are known to correlate closely with leaf nitrogen content in many crops, including citrus [53]. In each plot, 6 to 10 healthy, uniformly growing fruit trees were selected. For each tree, 3–4 leaves exposed to different orientations were sampled, and three measurements per leaf were taken at the tip, middle, and base, avoiding vascular veins. The average SPAD value per plot was used as a relative index of leaf nitrogen status.

In each experimental plot, five fruit trees exhibiting robust growth and uniform development were selected and labeled, yielding a total of 30 sample trees across all plots. During the fruit harvesting period (March to May 2025), fruits were harvested from the experimental trees for analysis of nutritional composition and fruit quality. For each tree, four fruits of comparable size, free from surface blemishes, and at uniform maturity were collected from the eastern, western, southern, and northern canopy positions. Upon collection, samples were transported to the laboratory for measurement and photographic documentation. The pulp was immediately extracted, flash-frozen in liquid nitrogen, and stored at −80 °C pending further analysis. The evaluated parameters included fruit quality grade (FQG), total soluble solids content (TSS, %), edible rate ratio (ER, %), total sugar (TS, g/100 mL), titratable acidity (TA, %) and vitamin C content (VC, μg/g). Fruit quality grade, categorized into three marketable grades: premium (1), first-grade (2), and second-grade (3), with grade 4 indicating unmarketable produce, was assessed based on fruit shape, color, diameter, and surface defects. TSS was determined using a handheld refractometer. Edible rate was defined as the percentage of edible weight to total weight. TS content was quantified via the Fehling’s reagent method, TA by indicator titration, and VC using the 2,6-dichlorophenolindophenol titration method [54].

In each area under different cover crop management models, 20 sample plants were randomly selected. The total number of fruits per plant was accurately recorded, and 10 fruits were randomly sampled from each plant to measure individual fruit weight. Plant yield was calculated by multiplying the total fruit count per plant by the average single-fruit weight, which was expressed in kilograms (kg). The mean yield per plant for each cover crop management model was determined by averaging the yields of the 20 sample plants.

#### 2.3.3. Statistical Analysis

The experiment was arranged in a treatment strip design with six treatments (CK, T1, T2, T3, T4, T5). Each treatment had 3 biological replicates (plots), for a total of 18 experimental units. The plot served as the experimental unit for all measurements. All statistical analyses were performed using R software (version 4.2.0). To determine whether leaf physiological trait status, fruit quality, soil physicochemical properties, and CO_2_ emission rates had differences under the five different cover crops treatments, a one-way analysis of variance (ANOVA) was first conducted. Following a significant ANOVA result (*p* < 0.05), a Tukey post hoc test was performed using the R package “multcomp” [55] to identify specific differences between treatment means. To explore the overall patterns of agroecosystem functioning traits under different ground cover management models, principal component analysis (PCA) was conducted using the “PCAtools” package [56] in R. Finally, the pairwise correlations between soil properties (including physicochemical properties and CO_2_ emission rates) and citrus traits (leaf physiological traits status and fruit quality) were examined using Mantel tests combined with Pearson’s correlation analysis. These analyses were performed using the “linkET” package [57] and “corrplot” package [58] in R. For all statistical tests, differences were considered statistically significant at *p* < 0.05.

## 3. Results

### 3.1. Effects of Different Cover Crop Management Models on Soil Properties

**Figure 2:**
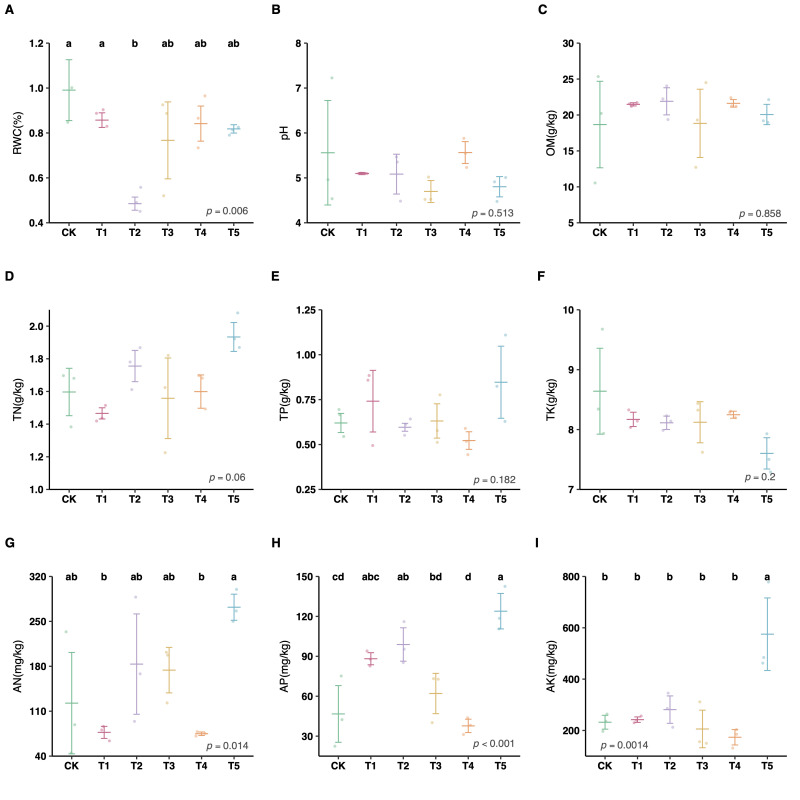
Effects of different cover crop management models on soil properties. (**A**) RWC: relative water content; (**B**) pH: soil acidity/alkalinity; (**C**) OM: organic matter; (**D**) TN: total nitrogen; (**E**) TP: total phosphorus; (**F**) TK: total potassium; (**G**) AN: available nitrogen; (**H**) AP: available phosphorus; (**I**) AK: available potassium. Different lowercase letters above the bars indicate significant differences between groups (*p* < 0.05). Panels without letters showed no statistically significant differences (*p* > 0.05).

The results of the one-way ANOVAs illustrated that four of the nine soil properties measured (RWC, AN, AP, AK) exhibited significant differences among the six treatment groups (including five cover crop management models and CK) (*p* < 0.05; Figure 2; see Table S1 for average values of the soil properties for the six groups). Results from Tukey tests indicated that T2 had the highest average value of RWC compared with the other cover crop management models (Figure 2A). T4 had the lowest average values of AN and AP among the groups (Figure 2G,H), and T5 had the highest average values of AN, AP, and AK (Figure 2G–I).

**Figure 3:**
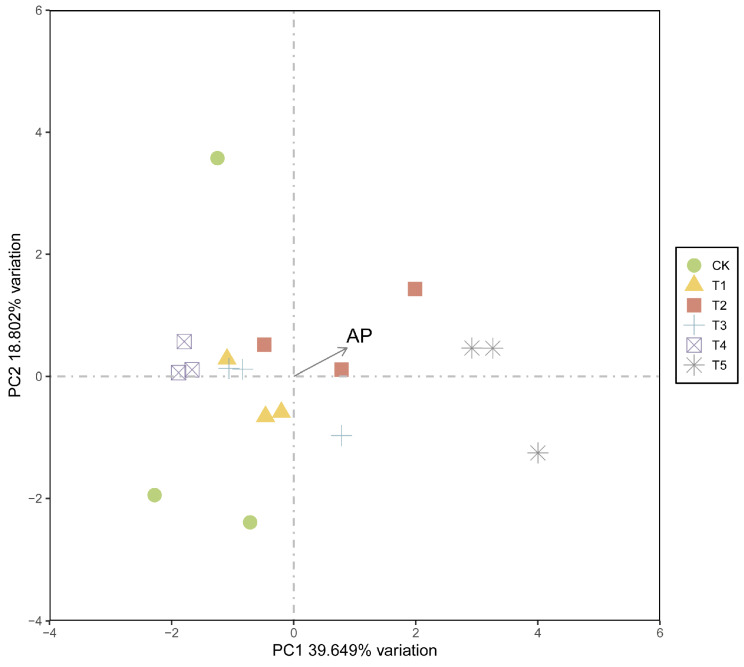
Principal component analysis (PCA) of the nine soil properties, showing their separation into two distinct regions. AP: Available Phosphorus.

The result of PCA showed that 18 samples of the 6 groups were separated into two distinct regions in soil properties (Figure 3). The first two principal components (PC1 and PC2) accounted for 39.649% and 18.802% of the total variance, respectively, cumulatively explaining 58.452% of the variation. The score plot revealed a distinct separation between treatments, particularly for T5, which was clustered far along the positive direction of PC1, indicating that T5 exerted the most profound impact on the soil profile compared to the control (CK) and other treatments. Furthermore, AP was identified as a key driver of this differentiation. The AP loading vector was positively correlated with both PC1 and PC2, aligning closely with the T5 group, which suggests that the T5 treatment significantly enhanced soil AP content relative to the other groups (Figure 2H, Table S1).

### 3.2. Effects of Different Cover Crop Management Models on Soil CO_2_

**Figure 4:**
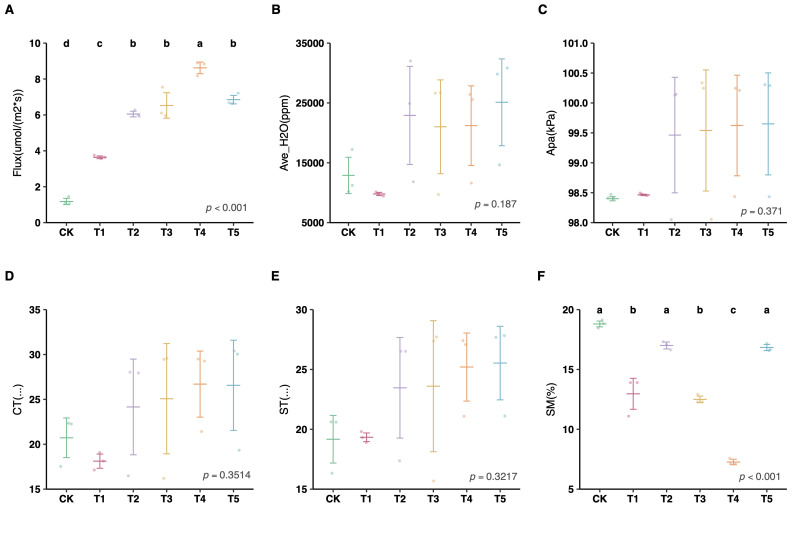
Effects of different cover crop management models on soil CO_2_. (**A**) Flux: soil CO_2_ flux; (**B**) Ave_H_2_O: average H_2_O content; (**C**) Apa: air pressure; (**D**) CT: chamber temperature; (**E**) ST: soil temperature; (**F**) SM: soil moisture. Different lowercase letters above the bars indicate significant differences between groups (*p* < 0.05). Panels without letters showed no statistically significant differences (*p* > 0.05).

The ANOVA statistical analysis revealed that soil CO_2_ flux (Figure 4A) and SM (Figure 4F) were significantly influenced by the treatments (*p* < 0.001). Specifically, mixed cover cropping (T5) and *Vicia villosa* (T4) dramatically increased soil respiration, while T4 resulted in the lowest SM. In contrast, other parameters such as CT and ST showed no significant variations across treatments (*p* > 0.05).

### 3.3. Effects of Different Cover Crop Management Models on Citrus Traits

**Figure 5:**
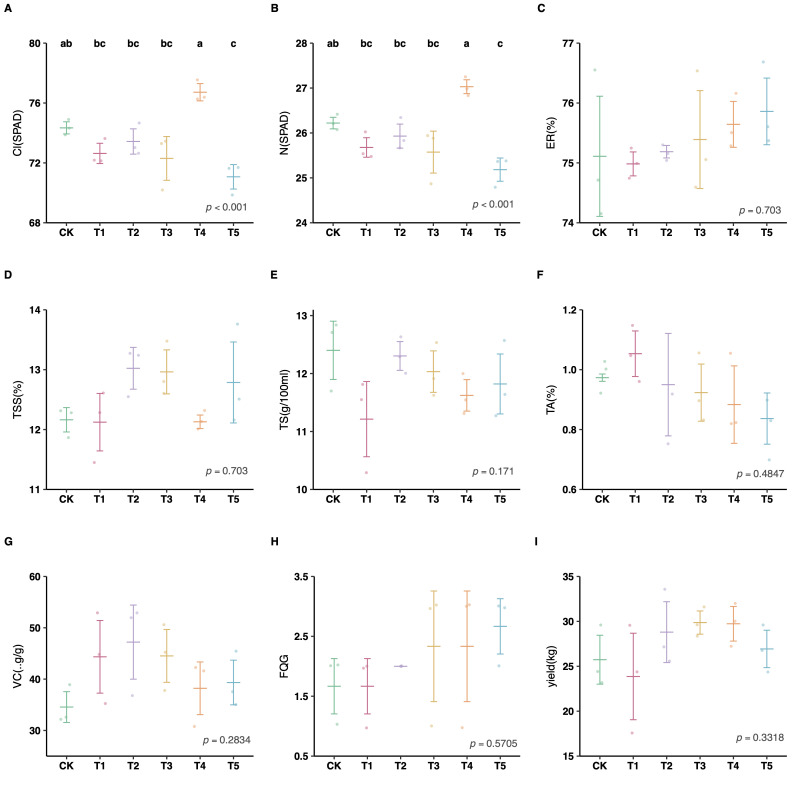
Effects of different cover crop management models on Citrus traits. (**A**,**B**): leaf physiological traits；(**C**–**I**): fruit quality and yield. (**A**) Cl: chlorophyll contents; (**B**) N: nitrogen content; (**C**) ER: eating rate; (**D**) TSS: total soluble solids; (**E**) TS: total sugar; (**F**) TA: titratable acid; (**G**) VC: vitamin C (ascorbic acid); (**H**) FQG: fruit quality grade; (**I**) Yield: total fruit yield. Different lowercase letters above the bars indicate significant differences between groups (*p* < 0.05). Panels without letters showed no statistically significant differences (*p* > 0.05).

Regarding leaf physiological traits, both Cl and N content exhibited highly significant responses to the treatments (*p* < 0.001) (Figure 5A,B). T4 (*V. villosa*) treatment resulted in the highest levels of leaf N and Cl among all groups, significantly outperforming the control. In contrast, T5 showed the lowest values for these traits, indicating a potential trade-off where improved soil phosphorus and water retention did not immediately translate into higher leaf nitrogen accumulation.

In terms of fruit quality and yield, no significant differences were observed among the treatments during the study period (*p* > 0.05). However, a slight upward trend was noted in yield (Figure 5I) and vitamin C content (Figure 5G) under cover crop treatments compared to the control. These results suggest that while cover cropping significantly improved soil properties and leaf physiological traits, the translocation of these benefits to fruit characteristics may require a longer duration to manifest statistically significant improvements.

### 3.4. Correlations Between Soil Properties, CO_2_ Flux, and Citrus Traits

**Figure 6:**
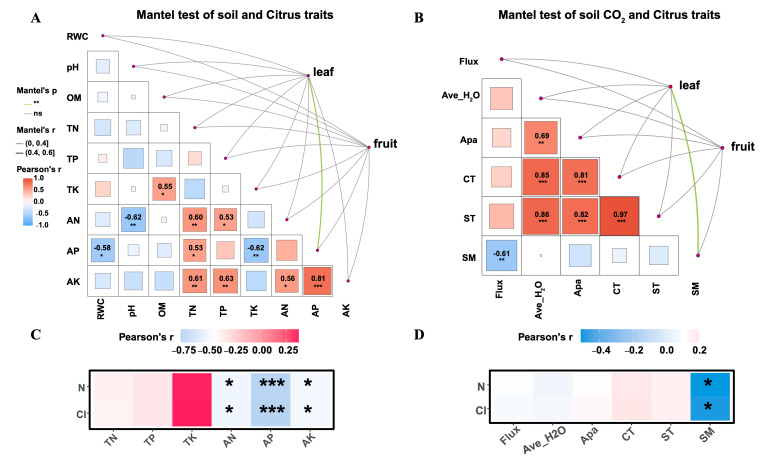
Relationships between soil physicochemical properties, CO_2_ flux, and citrus traits. (**A**,**B**): Relationships between soil properties and plant traits. (**C**,**D**): Influence of soil CO_2_ flux and citrus traits. Colored squares represent Pearson’s r. Line color/width represent Mantel’s significance (*p*) and strength (*r*). Ave_H_2_O: average H_2_O content. Significance: * *p* < 0.05, ** *p* < 0.01, *** *p* < 0.001.

The Mantel test highlighted that AP and SM were the most significant drivers of leaf traits (*p* < 0.01, indicated by green lines). Meanwhile, both Soil Properties and CO_2_ flux did not show correlations with citrus fruit traits (Figure 6A,C). Pearson correlation analysis revealed significant synergistic and antagonistic relationships between soil properties (Figure 6A). Notably, AK showed a strong positive correlation with AP (*r* = 0.81, *p* < 0.001) and TN (*r* = 0.61, *p* < 0.01; Figure 6A). Conversely, AP was negatively correlated with RWC (*r* = −0.58, *p* < 0.05; Figure 6A). Strong positive correlations were observed between ST and CT (*r* = 0.97, *p* < 0.001; Figure 6B), suggesting a tightly coupled thermal environment. SM, however, exhibited a significant negative correlation with soil CO_2_ flux (r = −0.61, *p* < 0.01; Figure 6B). The analysis of the correlation indices reveals distinct environmental drivers of Cl (Figure 6C,D). In terms of soil chemical properties, AP, AN, and AK showed highly significant negative correlations with Cl (AP: *r* = −0.761, *p* < 0.001; AN: *r* = −0.508, *p* = 0.031; AK: *r* = −0.5, *p* = 0.0343; Figure 6C), suggesting that elevated concentrations of available nutrients (AP, AN, and AK) may inversely impact these efficiency markers. Conversely, within the framework of soil and physical parameters (Figure 6D), SM emerged as the predominant factor, displaying a significant negative correlation with both the Cl (*r* = −0.5, *p* = 0.0342). This highlights the critical role of water content in modulating the dynamics of nutrient utilization efficiency.

## 4. Discussion

In this study, cover crop management models were found to influence soil properties in short time. By PCA, we found the most significant difference was AP among the five cover crop management models and CK. Moreover, the results of the ANOVAs indicated that cover crop management models could also influence citrus traits (including Cl, N) and soil CO_2_ (CO_2_ flux, and SM). Based on Pearson’s correlation, we found significant positive correlations of AP, AN, AK, and SM with leaf physiological traits. The results thus illustrate cover crop management models had a potential influence on citrus traits by changing soil properties.

### 4.1. Effects of Different Cover Crop Management Models on Soil Properties

A well-configured grassing system is proven to meaningfully change soil properties [16,17,18,59,60]. In citrus orchards, the various cover crop management models were found to change RWC, AN, AP and AK in soil (Figure 2). In particular, the cover crop with *Lolium perenne* (T2) remarkably decreased the RWC of soil (Figure 2A), while the cover crop with *Vicia villosa* (T4) remarkably decreased AN and AP (Figure 2B,C). The mixed cover crop combination (T5) was found to increase AK (Figure 2D). Mubvumba et al. discovered cover crops directly impacted the stored soil water of continuous wheat in Texas due to differ infiltration and water capture after termination [17]. They indicated that *L. perenne* had higher soil water use and availability and potential to improve stored soil water through greater infiltration and water capture after termination. Several studies have reported that cover crops directly improve the conditions of soil properties, notably by increasing the C–P and N–P ratios and C storage [59,60]. Our result demonstrated that *V. villosa* has a strong ability to absorb AP (Figure 2 and Figure 3) and AN (Figure 2). Because of this characteristic, *V. villosa* has the potential to reduce the impact of runoff on soil nutrient loss [61]. Mixed cover crop species potentially increase the orchard’s primary production with complementary resource use and, in turn, increase the production of litter and root exudates, which produces more soil nutrients [60]. Hence, we observed that mixed cover crop combination (T5) significantly increased AK in soil (Figure 2). Meanwhile, mixed cover crop species could improve the conditions of soil nutrients (e.g., significantly increased C–P, N–P, and carbon storage) to potentially enhance fruit yield [19,62].

### 4.2. Effects of Different Cover Crop Management Models on Soil CO_2_ Flux

The respiratory rate induced by root secretions in plants, especially leguminous plants, generally increases under drought stress [63]. In this study, we found that T4 significantly increased soil respiration with the lowest soil moisture content (Figure 4A,F); this implies that plants can alter root secretions to influence soil microorganisms and accelerate the decomposition of organic matter. Previous studies have shown that different cover crop management models affect CO_2_ flux, with elevated air temperatures enhancing microbial activity and thereby increasing CO_2_ emissions [64]. In addition, cover crops have been shown to directly influence soil temperature [65], which is consistent with the trend observed in the present study between the CK and cover crop treatments. Soil carbon flux is jointly driven by temperature and moisture, with temperature serving as the most consistently positive controlling factor [43,66]. Notably, T4 exhibited the highest CO_2_ flux despite having the lowest soil moisture content. A plausible explanation is that reduced soil moisture replaces water with air in soil pores, thereby improving soil aeration. Under such well-aerated conditions, root respiration is enhanced, leading to higher CO_2_ release [67]. Some studies have indicated that soil CO_2_ emissions follow a “peak-and-decline” pattern in response to soil moisture variation, where both excessively low and high moisture levels suppress CO_2_ emissions [68]. In contrast, mixed cover cropping enhances soil microbial functions by increasing the diversity of soil biological processes and catabolic metabolism [69]. This practice not only strengthens the soil microbiome but also promotes the cycling of carbon, nitrogen, and phosphorus [70], which aligns with the higher CO_2_ flux observed in T5 in this study (Figure 4). Furthermore, cover cropping has been reported to increase fungal biomass, and the C–N ratio of cover crop residues is an important variable explaining the dynamics of CO_2_ and N_2_O emissions [71,72]. Nevertheless, further monitoring data are needed to verify the patterns of soil carbon flux under different cover cropping systems in citrus orchards.

### 4.3. Effects of Different Cover Crop Management Models on Citrus

In general, soil properties play a key role in cover crops’ impact on fruit trees/commercial crops [59,60]. Cover crops can potentially enhance soil’s chemical and physical properties and contribute to biological processes in commercial crops *V. villosa* can increase Cl and N in leaves, enhance CO_2_ flux, and decrease SM for citrus trees (Figure 5). Under conditions of soil phosphorus enrichment, excess phosphorus can inhibit crop growth and development by disrupting metabolic balance, thereby weakening photosynthetic efficiency [73] and chlorophyll synthesis [74,75,76]. Based on Pearson’s correlation analysis, AP had a significant negative relationship with Cl in citrus leaves (Figure 6). This implies that the ability to absorb AP of *V. villosa* could improve chlorophyll synthesis of citrus.

We did not find that the five cover crop management models related to citrus yields , despite some studies reporting that cover crops often result in higher yields of commercial crops [77,78]. In many cases, there was zero or negative impact of cover crops on crop yields [79,80]. This is because cover crops may deplete stored soil moisture [17] or their impact on subsequent crop yields can take many years to manifest.

### 4.4. Strengths and Limitations of This Study

This study provides a comprehensive evaluation of various cover crop management models in citrus orchards, integrating soil health indicators with plant physiological performance. A major strength lies in the identification of *Vicia villosa* as an optimal cover crop for nutrient uptake and its mechanistic linkage to soil moisture and phosphorus availability. However, several limitations should be acknowledged. Firstly, the study reflects a short-term observation (two years). Given that the ecological benefits of cover cropping and the physiological feedback of perennial fruit trees often accumulate over time, a longer-term monitoring effort is required to assess the stability of these benefits. Secondly, the lack of significant impact on fruit yield and quality during the study period suggests a ‘lag effect’ common in orchard management. Lastly, while our results offer practical guidance for citrus management in the subtropical Three Gorges Reservoir area, the generalizability of these findings to other soil types and climatic conditions warrants further investigation. We acknowledge that quantitative measurements of cover crop biomass and soil coverage were not conducted in the present study, although visual observations confirmed vigorous and uniform growth covering the entire plot area. Future studies should include such measurements to better establish the relationship between cover crop growth characteristics and soil property changes.

## 5. Conclusions

This study demonstrates that orchard cover crop management models can rapidly modulate soil properties, carbon dynamics, and citrus physiological traits within a short period. Our findings identify *Vicia villosa* (T4) as the most effective management model for enhancing orchard ecosystem functions. Specifically, T4 exhibited a superior capacity for available nitrogen (AN) and phosphorus (AP) uptake. Notably, the reduction of surplus soil AP by T4 was found to alleviate nutrient interference, thereby significantly promoting leaf chlorophyll synthesis and nitrogen accumulation in citrus trees. Although soil flux increased under T4 due to altered soil temperature and aeration, the rapid optimization of soil fertility and tree physiological vigor provides a robust scientific basis for sustainable orchard intensification. While fruit yield remained stable during the two-year experimental period—likely due to the time-lag effect inherent in perennial crops—this study highlights that mixed cover cropping and Fabaceae species (*V. villosa*) are critical strategies for achieving long-term ecological and productivity synergies in citrus cultivation.

## Data Availability

All data supporting the conclusions of this article are provided within the article (and its additional files).

## Supplementary Materials

The following supporting information can be downloaded at: https://www.mdpi.com/article/10.3390/plants15101479/s1, Table S1: Data of soil properties, soil CO_2_, leaf physiological traits, fruit quality and yield among the five cover crop management models and CK.
